# Effect of Capacity to Undertake Instrumental Activities of Daily Living on Entry to Aged Residential Care in Older People With Heart Failure

**DOI:** 10.3389/fmed.2020.00386

**Published:** 2020-07-31

**Authors:** Hamish A. Jamieson, Rebecca Abey-Nesbit, John W. Pickering

**Affiliations:** ^1^Department of Medicine, University of Otago, Christchurch, New Zealand; ^2^Older Persons Health, Burwood Hospital, Canterbury District Health Board, Christchurch, New Zealand

**Keywords:** heart failure, instrumental activities of daily living (IADL), aged residential care, older people, InterRAI, resilience

## Abstract

**Background:** Heart failure is a common condition in older people with complex medical needs. A key factor in resilience after heart failure is the capacity to perform the instrumental activities of daily living (IADLs). Knowing the association between capacity to perform IADLs and entry into aged residential care will help health professionals plan interventions that will allow older people to remain independent longer.

**Methods:** We analyzed the association between the capacity to perform eight IADLs and entry into ARC. Participants included New Zealanders aged ≥65 years with a diagnosis of heart failure, and who had an InterRAI 9.1 Home Care assessment between July 2012 and June 2018. A multivariable competing risks regression model for entry to ARC with death as the competing risks was used to establish sub-hazard ratios (SHR) for IADL capacity. Co-variates included demographic variables, co-morbidities, living arrangements, cognitive performance, depression, timed walk, alcohol use, smoking, activities of daily living, recent hospitalization and history of falls.

**Results:** There were 13,220 participants with heart failure who were followed for a median 1.69 (0.70–3.17) years. There were 3,177 (24.0%) participants who entered aged residential care and 5,714 (43.2%) who died without having first entered residential care. Overall capacity to perform specific IADLs was “very poor” for housework (85.5%), shopping (68.0%), stairs (61.7%), meal preparation (53.0%), and transportation (52.2%). In the multivariable model, compared to adequate capacity (the reference) poorer capacity for managing finance, managing medications, meal preparation and transport were all associated with increased risk of entering aged residential care, with SHR from 1.05 to 1.18. Overall, the IADL capacity explained ~10% of the risk of entering aged residential care.

**Conclusion**: Capacity to perform IADL is a key factor in maintaining resilience in older people with heart failure. Capacity to manage finances, transport and medications, prepare meals, and transport oneself with minimal supervision could reduce the risk of entry into aged residential care. Developing early interventions and support for people with poor capacity to perform their IADL may help reduce admission into aged residential care.

## Introduction

Heart failure is a common condition affecting ~38 million people worldwide (1). It is most common in adults more than 60 years in age. The prevalence of heart failure is increasing (2, 3) with an estimated 5–10 new diagnoses per 1,000 persons per year (4).

Heart failure has serious impacts on older people including high rates of hospitalization. Generally, the prognosis for heart failure patients is poor with many individuals having reduced life expectancy compared to those without heart failure (4). After a heart failure exacerbation, the 30-day mortality is ~10–20% (4). A heart failure event may be the trigger for some older people to enter aged residential care facilities (ARC) because they are or believe they are unable to manage at home. However, there may be other factors contributing to the decision to enter ARC. ARC is costly, the daily cost per resident can range from $148.33 to $162.30 (NZD) (5). As of 2013, there were 31,899 people living in ARC facilities in New Zealand (6).

The New Zealand interRAI-Home Care (HC) is a comprehensive clinical assessment used to assess community-dwelling older adults to help provide tailored home care services. It captures diagnoses of heart failure and provides a unique opportunity to investigate factors that may be associated with entry to ARC. Extensive training is given to interRAI assessors to ensure that all assessments are undertaken with the same standard of quality (7). As well as providing information useful for individually tailored care plans for older people, the interRAI data can be used for larger scale research.

Instrumental Activities of Daily Living (IADL) are self-care activities that often require more complex interactions than Activities of Daily Living (ADL) (8). For example, ADLs include bathing, personal hygiene, and bed mobility, whereas IADLs are more complex items such as managing medications, phone use, and meal preparation. IADLs are key factors in resilience of older people. A key factor contributing to resilience after heart failure is the capacity to perform the instrumental activities of daily living (IADLs). Knowing the association between capacity to perform IADLs and entry into aged residential care (ARC) would help health professionals plan interventions that allow older people to remain at home longer. The aim of this study was to identify within older people with heart failure any associations between capacity to perform IADLs and entry to ARC.

## Materials and Methods

### Design

This was a prospective observational study of routinely collected health data.

### Participants

**Figure 1:**
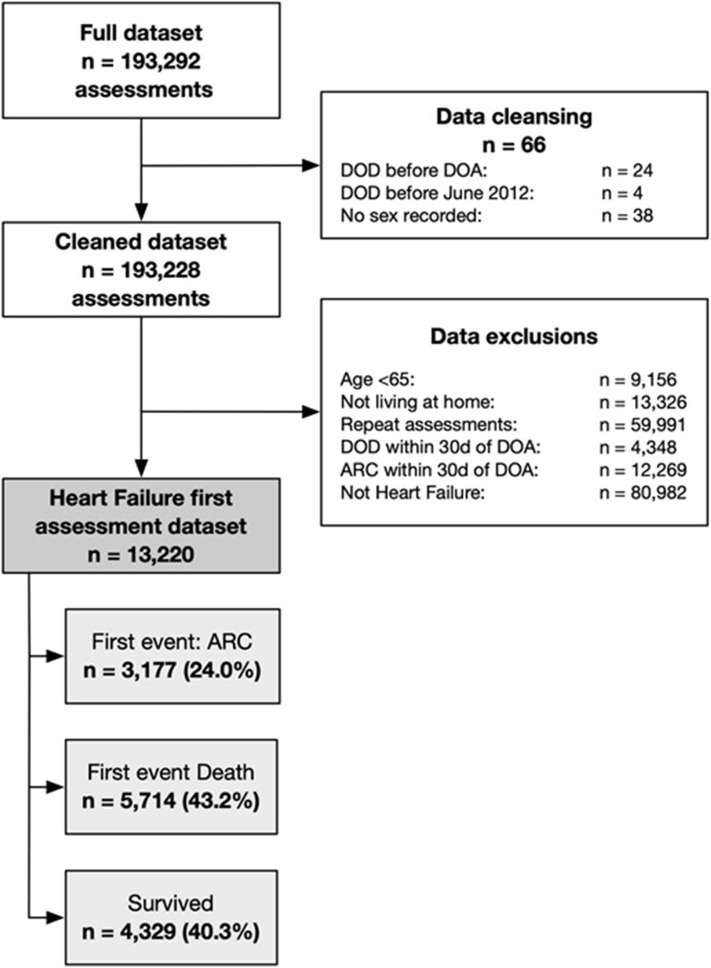
Participant selection criteria.

Participants consisted of individuals aged 65 years and older who underwent an interRAI-HC assessment between 1 July 2012 and 30 June 2018 and who had received a diagnosis of heart failure in the interRAI. All participants consented for their data to be used for research purposes. The interRAI-HC assessment is compulsory for all older people wanting to receive publicly funded health care services, including funding to stay in aged residential care. Exclusion criteria included death or entry into ARC within 30 days of assessment, living in a non-home setting at the time of assessment, and any repeat assessments. Figure 1 details the participant selection criteria. Each participant had a minimum follow-up of 30 days, and the study period ending was 30 July 2018.

### Data Collection

Anyone who receives healthcare in New Zealand is assigned a unique identification number known as their national health index number (NHI) number (9). Encrypted NHI numbers were used to link interRAI-HC assessments with mortality and ARC entry data. New Zealand's Technical Advisory Services provided interRAI-HC records of all individuals who consented for their data to be used for research and planning purposes. ARC admission dates were provided by The Ministry of Health using its Contracted Care Payment System database. The interRAI database only contains publicly funded ARC residents which is ~90% of all ARC residents in New Zealand. The other 10% are privately funded individuals who may or may not have completed an interRAI-HC assessment before entering ARC. Mortality information was provided by The National Mortality Collection Register.

In section G of the interRAI-HC 9.1 assessment tool there are two types of IADL assessment. The first is for self-performance of routine activities during the previous 3 days and the second is for capacity based on presumed ability to carry out the activity as independently as possible. For this analysis we have chosen to use the capacity scores only to avoid the issue of large amounts of missing data due to no activity taking place in the previous 3 days. There are seven IADL activities, namely; meal preparation, ordinary housework, managing finances, managing medications, phone use, managing a full flight of stairs, shopping performance, and managing transportation. Functional status for each IADL is graded on a scale: Independent, Setup help only, Supervision (oversight/cuing), Limited assistance (help on some occasions), Extensive assistance (help throughout the task but performs ≥50% of the task on own), Maximal assistance (help throughout the task but performs <50% of the task on own), and Total dependence. To avoid overfitting or spurious results due to smaller numbers in any grade, for the competing risks model we grouped Independent and Setup help only as “Adequate”; Supervision and Limited Assistance, as “Poor”; and Extensive Assistance, Maximal Assistance, and Total dependence as “Very Poor.”

### Statistical Analysis

Descriptive information of variables of interest were reported. For categorical variables data are presented as *n* (%); for normally distributed quantitative variables as mean and standard deviation; and for non-normally distributed variables as median and the lower quartile-upper quartile range. Correlations are Spearman r.

A multivariable competing-risks regression model was used to assess the association between IADLs and ARC entry. A competing risk model was required to account for the substantial number of people who die before they may otherwise have entered ARC. This makes death a competing risk. The effects of variables on the likelihood of participants entering ARC were reported as sub distribution proportional hazard ratios (SHR). The Fine-Gray method was used to calculate SHRs with the *cmprsk* package in R (10). A SHR >1 indicates an increased risk of entering ARC relative to the reference (e.g., SHR of 1.5 indicates a 50% increase in risk), whereas a SHR <1 indicates a decreased risk (e.g., SHR of 0.67 indicates a 1/0.67 = 1.5 or 50% decrease in risk).

To assess the relative importance of each variable, we calculated the percentage contribution of variable to the model. We used the chi-square statistic minus the number of degrees of freedom for that variable relative to the chi-square minus the number of degrees of freedom for all the model; we present these graphically (11).

The STROBE guidelines (www.strobe-statement.org) were used to guide this report. All calculations were performed in R version 3.5.2 (12). Ethics approval was obtained from the Ministry of Health, Health and Disability Ethics Committee (14/STH/140/AM07).

## Results

There were 13,220 participants with heart failure of whom the first event was entry into ARC for 3,177 (24.0%) and death for 5,714 (43.2%), and 4,329 (40.3%) survived until the end of the study period without entering ARC (Figure 1). Participants were followed for a median 1.69 [interquartile range: 0.70–3.17, maximum: 5.85)] years.

More females (56.9%) than males were assessed (Table 1). The mean (SD) age was 83.1 years (7.3 years). The population comprised 10.0% Māori, 3.0% Pacific peoples and 84.4% New Zealand Europeans. At the time of assessment, a little under half the participants (48.7%) were living alone. Hospitalization in the previous 90 days was common (44.3%), as was Dyspnea (72.7%), some Fatigue (80.3%), Coronary Heart Disease (CHD: 53.6%), Chronic Obstructive Pulmonary Disease (COPD: 26.8%) and Diabetes Mellitus (DM: 26.3%). A full table of the variables of interest can be found in Table S1.

**Table 1:** Demographics and IADL frequencies and Sub Hazard Ratios (SHR) for entry to ARC from multivariable competing risks models.

	**Survive**	**ARC**	**Death**	**Multivariable**
	**(*n* = 4,329)**	**(*n* = 3,177)**	**(*n* = 5,714)**	**SHR for ARC**
**Age**				
Mean (SD)	81.1 (7.47)	84.5 (6.68)	83.7 (7.30)	1.02 (1.01 to 1.02)
**Sex**				
Female	2,633 (60.8%)	1,949 (61.3%)	2,937 (51.4%)	1 Reference
Male	1,696 (39.2%)	1,228 (38.7%)	2,777 (48.6%)	0.85 (0.76 to 0.93)
**IADL Meal Preparation Capacity**				
Adequate	1,762 (40.7%)	858 (27.0%)	1,438 (25.2%)	1 Reference
Poor	748 (17.3%)	579 (18.2%)	829 (14.5%)	1.18 (1.06 to 1.29)
Very poor	1,819 (42.0%)	1,740 (54.8%)	3,447 (60.3%)	1.05 (0.94 to 1.16)
**IADL Housework Capacity**				
Adequate	250 (5.8%)	117 (3.7%)	184 (3.2%)	1 Reference
Poor	549 (12.7%)	328 (10.3%)	494 (8.6%)	0.96 (0.75 to 1.17)
Very poor	3,530 (81.5%)	2,732 (86.0%)	5,036 (88.1%)	0.97 (0.78 to 1.16)
**IADL Finance Capacity**				
Adequate	2,440 (56.4%)	1,193 (37.6%)	2,390 (41.8%)	1 Reference
Poor	674 (15.6%)	575 (18.1%)	879 (15.4%)	1.17 (1.06 to 1.28)
Very poor	1,215 (28.1%)	1,409 (44.4%)	2,445 (42.8%)	1.15 (1.04 to 1.26)
**IADL Managing medications Capacity**				
Adequate	2,635 (60.9%)	1,437 (45.2%)	2,807 (49.1%)	1 Reference
Poor	804 (18.6%)	775 (24.4%)	1,230 (21.5%)	1.14 (1.04 to 1.25)
Very poor	890 (20.6%)	965 (30.4%)	1,677 (29.3%)	1.16 (1.05 to 1.28)
**IADL Phone use Capacity**				
Adequate	3,739 (86.4%)	2,495 (78.5%)	4,426 (77.5%)	1 Reference
Poor	231 (5.3%)	231 (7.3%)	467 (8.2%)	0.83 (0.69 to 0.98)
Very poor	359 (8.3%)	451 (14.2%)	821 (14.4%)	1 (0.87 to 1.14)
**IADL Stairs Capacity**				
Adequate	1,198 (27.7%)	687 (21.6%)	1,064 (18.6%)	1 Reference
Poor	722 (16.7%)	545 (17.2%)	853 (14.9%)	1.06 (0.94 to 1.17)
Very poor	2,409 (55.6%)	1,945 (61.2%)	3,797 (66.5%)	1.06 (0.96 to 1.15)
**IADL Shopping Capacity**				
Adequate	1,306 (30.2%)	545 (17.2%)	898 (15.7%)	1 Reference
Poor	561 (13.0%)	364 (11.5%)	562 (9.8%)	1.07 (0.93 to 1.21)
Very poor	2,462 (56.9%)	2,268 (71.4%)	4,254 (74.4%)	1.08 (0.96 to 1.21)
**IADL Transportation Capacity**				
Adequate	2,013 (46.5%)	957 (30.1%)	1,708 (29.9%)	1 Reference
Poor	521 (12.0%)	441 (13.9%)	638 (11.2%)	1.18 (1.06 to 1.31)
Very poor	1,795 (41.5%)	1,779 (56.0%)	3,368 (58.9%)	1.04 (0.93 to 1.14)

**Figure 2:**
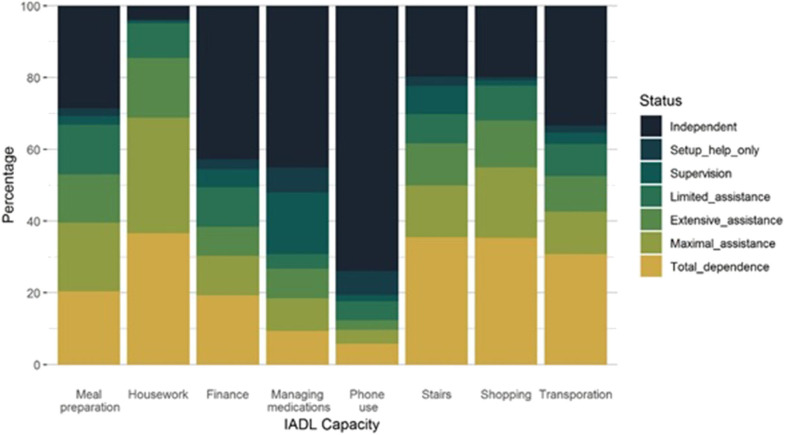
IADL capacity and independence level frequencies.

**Figure 3:**
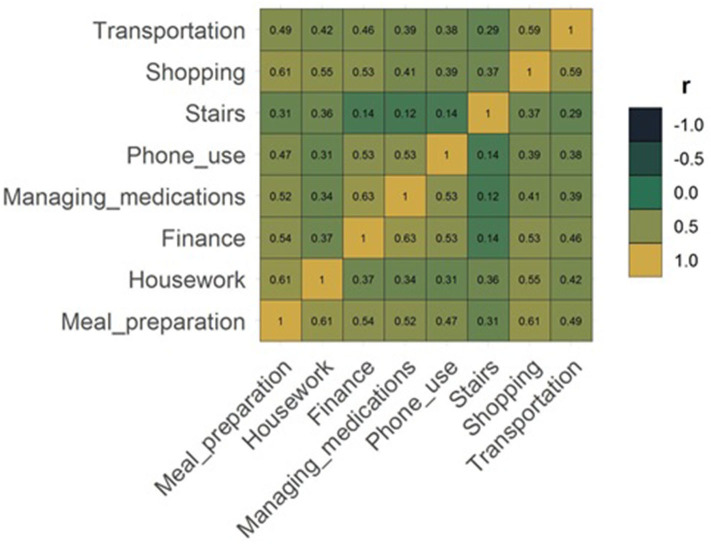
Correlation matrix between different IADLs.

When all IADL participant capacities are considered (in all participant categories: Survive, ARC, Death), the most independent was phone use and least housework (Figure 2). The overall capacity for housework was *very poor* for 85.5% of the participants. Also, *very poor* were capacity for shopping (68.0%), stairs (61.7%), meal preparation (53.0%), and transportation (52.2%). The capacity was *adequate* for phone use for 80.6% and for managing medications for 52.0% of participants (Table 1). Many of the IADLs were moderately correlated with each other, except for the capacity to use stairs which was only weakly correlated with the other IADLs (Figure 3). The strongest correlations were between transportation and shopping, shopping and meal preparation, and meal preparation and housework.

**Figure 4:**
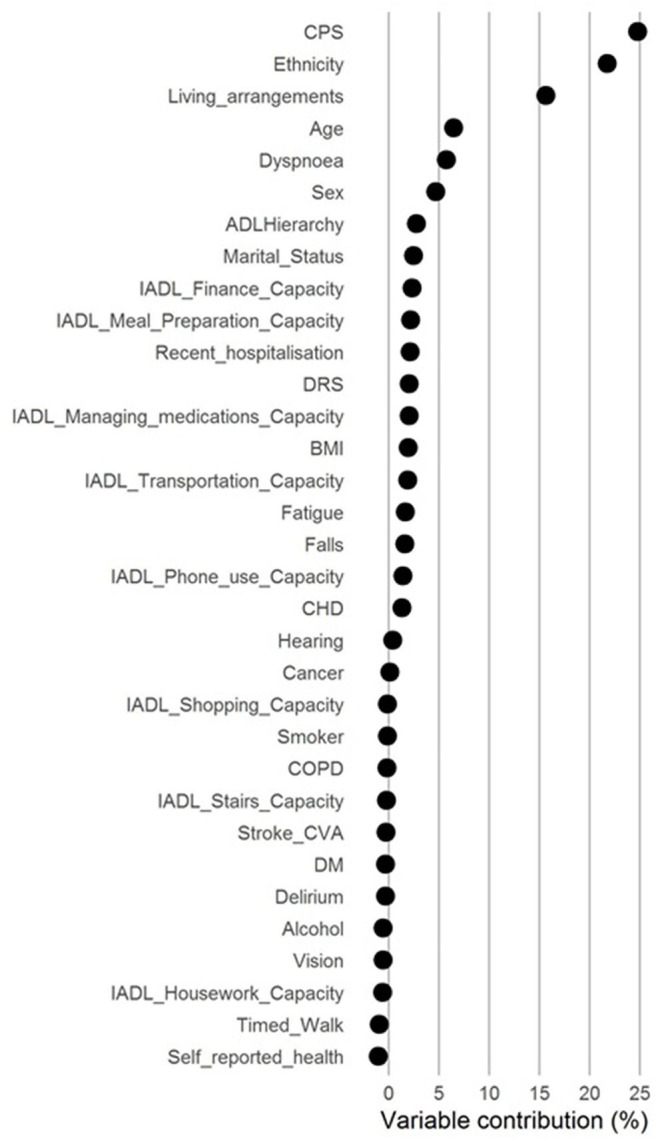
Variable percentage contribution to the regression model.

In the multivariable model for entry to ARC, compared to *adequate* capacity (the reference) poorer capacity for managing finance, managing medications, meal preparation and transport were all associated with increased risk of entering ARC (Table 1). The point estimates of the SHR were greatest for poor meal predation preparation capacity and poor transportation capacity indicating an 18% increased risk. These increased risks were slightly greater than those in the multivariable model for a recent fall (12% increased risk for fall within previous 30 days; Table S1) or depression (13% increased risk for depression indicated on the Depression Rating Scale; Table S1). The IADL increase in risk were less than for decreased cognitive performance which ranged from a 26% increased risk for minimal loss of cognitive performance on the Cognitive Performance Scale to 118% for severe loss of cognitive performance (Table S1). Each IADL contributed independently between 0 and 3% of the overall risk of entering ARC and combined ~10% of the overall risk (Figure 4).

## Discussion

This study confirmed that capacity to perform instrumental activities of daily living is associated with entry into ARC. After accounting for multiple confounding factors, and compared to *adequate* capacity (the reference), poorer capacity for managing finance, managing medications, meal preparation and transport were all associated with increased risk of entering ARC. Each IADL contributed independently between 0 and 3% of the overall risk of entering ARC.

### Instrumental Activities of Daily Living and Entry Into Aged Residential Care

While all IADLs were associated with entry into ARC, managing finances and managing medications contributed more highly to ARC entry than several other variables. Both difficulty managing finances and medications are associated with cognitive impairment (13–15), which can also lead to entry into ARC (16, 17). Additionally, poor medication management has been previously reported associated with entry into ARC (18). Medication management is particularly important as a mismanagement of medications could lead to an overdose or missing a needed dose of medication could lead to adverse health effects. HF patients have a high medication load and so mismanagement of medications carries a particularly high risk.

The IADL with the second highest contribution to ARC entry was meal preparation. When an individual has problems with meal preparation, they may be less likely to eat enough food each day. This can lead to health issues such as malnutrition and severe weight loss. Anyone having issues with meal preparation may be more likely to enter ARC where their meals will be prepared for them.

While our primary interest was ARC entry and death was treated as a competing risk, we note that for all participants IADL capacity was poorer in those who died compared to those who survived without entry to ARC. Very poor IADL (meal preparation, stair use, transportation) capacity was higher in those who died than those who entered ARC.

### Limitations With the InterRAI

This research was based on a large national database of comprehensive clinical assessments, the data was linked with other health outcomes allowing for extensive adjustment of confounders and more accurate health information. However, there are some limitations. The ARC database does not include everyone who entered ARC, rather only those who receive publicly funded care. Approximately, 10% of people living in ARC will not be recorded and any fundamental differences between this 10 and the 90% receiving publicly funded care is unknown. There may have been some loss of follow up information if people leave New Zealand, when this happens there are no ARC entry or mortality records for that individual. To avoid double counting individuals only first homecare assessments were used, however, this means any later assessments where the individual might have more complex health needs were not considered.

This study was completed using information from a New Zealand population of people over the age of 65 years with complex health care needs who were being considered for homecare services. The findings, therefore, may not be generalizable to a healthier population of people nor for an international audience. Nevertheless, the results could be used to inform studies in these populations.

## Conclusion

With the growing aging population, it is important to understand resilience and drivers of entry into residential care. Capacity to perform IADLs is a key factor for those with HF. Interventions and services that assist with the management of IADLs, especially finances and medications may delay entry into aged residential care.

## Data Availability Statement

The datasets generated for this study will not be made publicly available. The data is held by Technical Advisory Services in New Zealand who do not allow for the distribution and sharing of the data. Anyone who wishes to gain a copy of the dataset must apply to Technical Advisory Services.

## Ethics Statement

The studies involving human participants were reviewed and approved by Health and Disability Ethics Committee 14/STH/140/AM07. The patients/participants provided their written informed consent to participate in this study.

## Author Contributions

HJ conceived the study and its design, acquired the funding, oversaw the study, contributed to interpreting the data and writing the paper, and approved the manuscript for submission. RA-N contributed to data analysis and writing the paper. JP led the data analysis and its interpretation. All authors contributed to the article and approved the submitted version.

## Conflict of Interest

The authors declare that the research was conducted in the absence of any commercial or financial relationships that could be construed as a potential conflict of interest.
